# Nanocrystals in Dermal Drug Delivery: A Breakthrough for Enhanced Skin Penetration and Targeted Skin Disorder Treatments

**DOI:** 10.3390/pharmaceutics16121561

**Published:** 2024-12-06

**Authors:** Ahmed S. Alnaim

**Affiliations:** Department of Pharmaceutical Sciences, College of Clinical Pharmacy, King Faisal University, Al-Ahsa 31982, Saudi Arabia; asaalnaim@kfu.edu.sa

**Keywords:** dermal drug delivery, nanocrystals, bioavailability, drug loading capacity, skin disorders

## Abstract

One of the major challenges in dermal drug delivery is the adequate penetration of the active compound into the skin without causing any skin irritation and inflammation. Nanocrystals (NCs) are nanoscale particles, and their sizes are below 1000 nm. NCs are made up of drug particles only, which are used to improve the aqueous solubility and bioavailability of poorly water-soluble drugs. NCs are typically prepared either by bottom-up or top-down techniques. The advantages of using NC-based formulations in enhancing dermal drug delivery include increased drug loading capacity, easier and deeper penetration into the skin tissue, and increased passive diffusion. NC-based formulations with the capacity of enhanced dermal drug delivery can be effectively used to treat a wide range of skin disorders, including melanoma, inflammation, psoriasis, acne vulgaris, bacterial infections, fungal infections, eczema, skin aging, herpes simplex virus infections, skin manifestations of tick bites, frostbite-related infections, hyperpigmentation, and diabetic foot ulcer. In this review, major challenges in dermal drug delivery across the skin barrier, mechanism of action of dermal NCs, advantages of using NCs in enhancing dermal drug delivery, NC preparation methods, and applications of NCs in the treatment of various skin disorders have been discussed.

## 1. Introduction

The large skin surface area (approximately 20 square feet) makes it a potential route to deliver drugs [1]. In addition to oral drug delivery, dermal drug delivery (DDD) is a constantly growing and major market in the pharmaceutical field. In addition, because of the growing interest in DDD and the higher occurrence of skin injuries or disorders, there is a need for the design and development of novel and effective topical formulations that can provide solutions to the current problems faced in DDD. One of the major challenges in DDD is the adequate penetration of the active compound through or into the skin without causing any skin irritation and inflammation. For example, penetration enhancers used in DDD are responsible for causing skin rashes, irritation, and inflammation [2]. The topical formulation ideally should deliver the active pharmaceutical ingredient as well as restore or maintain the functions of the skin barrier. The term corneotherapy is used to define the therapeutic pathway that effectively delivers the drugs as well as restores or protects skin barrier function in order to enhance the efficacy of the treatment and patient compliance [3,4,5]. Most commonly, DDD through a cream formulation is used to treat skin disorders [6]. The characteristics of inflammatory skin conditions include damage to the integrity of the stratum corneum (SC), which can mediate epicutaneous therapies [7]. However, each skin condition has a distinct extent of impaired skin integrity, which makes the standard skin care approaches challenging.

Furthermore, the effectiveness of direct penetration through the SC is crucial to determine the precise properties of topical medications, as the chemical structures of drugs affect solute interactions with the lipid bilayers [8]. The integration of medications into vehicles, including nanocarriers, is envisioned to enhance the penetration of topical medications through intact or disrupted skin barriers. Targeted dosages of the drugs are formulated to ameliorate the efficacy of drugs to cure or improve the affected skin areas with minimum side effects. A range of carrier systems have recently been developed as per the physical and chemical properties of drugs of interest. Such carrier systems include nano-emulsions, liposomes, and polymer-based particles [9,10,11]. Unfortunately, these carrier systems have poor success rates owing to the inadequate drug load [12]. In addition, a large portion of newly developed drug candidates suffer from a very low solubility, which significantly affects their druggability unless they are being reformulated [13]. As compared to classic solubilization techniques, advances in nanotechnology have greatly opened novel approaches to improve solubility, including cyclodextrin complexation, co-crystal formation, and salt formation. Out of different drug delivery systems, nanocrystals (NCs) have revolutionized and improved the solubility and bioavailability of poorly soluble drugs [13,14].

NCs are nanosized drug particles, and the process of nanocrystallization is used to improve the aqueous solubility and bioavailability of poorly water-soluble drugs [15]. NCs are entirely made up of drug particles with sizes below 1000 nm, which are conventionally produced either through top-down or bottom-up approaches. Physicochemical properties are altered due to the smaller particle sizes, which further results in increased kinetic solubility. Moreover, an increase in the particle curvature of NCs results in elevated dissolution pressure, which eventually raises solubility [16]. The elevated surface area and solubility ameliorate the dissolution velocity of poorly soluble drugs, and the increased solubility leads to an elevated concentration gradient, which ultimately mediates passive diffusion via biological membranes. Moreover, nanosized particles have an elevated adhesiveness to membranes since the surface-to-volume ratio elevates as the size reduces [16]. This phenomenon is beneficial in terms of drug delivery since a prolonged retention time at the absorption site will mediate the penetration of active ingredients [17]. These advantageous features are already being used in the formulation of oral drugs [15,18,19]. Indeed, the beneficial properties of NCs can also be utilized for DDD since skin penetration in the case of DDD is typically driven via passive diffusion [16,20]. Although no commercial NC-based formulations are available in the market as dermal therapy, numerous cosmetic products based on the NC principle are currently available in the market [21]. In this review, major challenges in DDD across the skin barrier, mechanism of action of dermal NCs, advantages of using NCs in enhancing DDD, NC preparation methods, and applications of NCs in the treatment of various skin disorders have been discussed.

## 2. Routes of Drug Penetration and Challenges in Dermal Drug Delivery Across Skin Barrier

**Figure 1:**
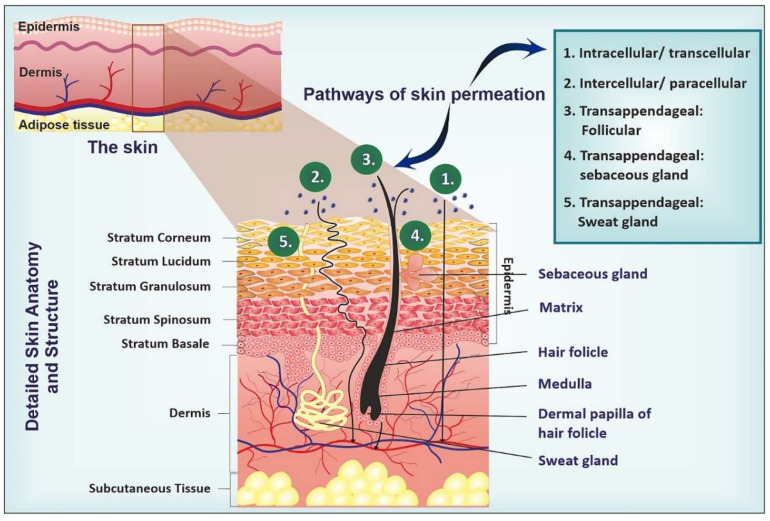
Structure of skin and penetration routes of drugs administered across the skin. Reproduced with permission from Elsevier, Reference [29].

Skin is the body’s largest organ, which is composed of 16% of total body weight and covers around 1.8 m^2^ of body area [22]. The skin has three different layers, including the epidermis, dermis, and hypodermis (Figure 1). The structure and composition of these layers are different [23]. The dermis layer is made of a dense network of fibers, including elastin and collagen. The drugs that reach the dermis later can also be passed into the systemic circulation. The outermost layer of skin is the epidermis, which is further divided into the viable epidermis (a living hydrophilic layer) and SC (a hydrophobic layer) [24]. SC is also known as a horny layer, which is composed of dead cells [22,25]. SC represents the utmost challenge for active drug penetration through the viable skin. SC is made of 15–20 layers of corneocytes entrenched in a lipid matrix, and this matrix is composed of cholesterol, free fatty acids, and ceramides. Since SC is located in the outermost layer of the epidermis, therefore this layer serves as a barrier that restricts the therapeutic efficacy of numerous drugs via serving as a barrier for drug molecules [12,26]. Drugs pass across these barriers through specific routes, including transcellular (intracellular), intercellular (paracellular), and transappendageal routes (Figure 1). Typically, lipophilic or hydrophobic molecules penetrate through the corneocytes via the transcellular route, while hydrophilic drugs preferentially penetrate through the SC via the intercellular route. On the other hand, drugs usually cross the skin appendages (including hair follicles, sebaceous glands, and sweat glands) through the transappendageal route [27,28]. Indeed, the uniquely intact skin structures play a role as a major barrier for most of the drugs. Therefore, several novel techniques have been designed and developed for effective DDD, including NCs.

## 3. Nanocrystals (NCs)

NCs are nanoscale particles, and their sizes are below 1000 nm [30]. NCs are made up of drug particles only, which are stabilized by stabilizers and kept dispersed in order to avert their aggregations. The common stabilizers that are used in the formulation of NCs include polymeric, non-ionic, and ionic stabilizers [15]. NC-based formulations can greatly enhance dissolution rate, drug release rate, surface adhesion, saturation solubility, and dermal bioavailability of the drug. In DDD, physicochemical characteristics (including size, shape, and surface area) of nanostructured particles play an important role in regulating the skin permeation process. The smaller size of NCs largely enhances drug delivery across the skin barriers. Moreover, NCs exhibit an inherent increased surface area-to-volume ratio, which facilitates enhanced interaction of drugs with the SC. This enhanced interaction is beneficial in overcoming the challenging barrier characteristics of the SC, which further mediates more expedited and efficient transcutaneous delivery of active drugs through the viable skin. Modification of the morphology or shape of NCs also has a significant contribution to increasing the DDD of active drugs through the skin barrier. It has been observed that anisotropic NC shapes, including nanowires or nanorods, have an elongated geometry that offers an outstanding benefit in crossing intercellular spaces of the SC [31]. It was reported that NCs of curcumin did not show any erythema or edema formation and had an average skin irritation index value of zero [32]. However, NCs with sharp edges or irregular shapes may cause skin irritation.

Top-down or bottom-up approaches are used to prepare NCs. NCs can also enhance the absorption rate of poorly water-soluble drugs in the skin. In a study, it was observed that as compared to the lutein microcrystals, NCs (nanosuspension) showed enhanced saturation solubility and enhanced skin penetration [33]. Since NCs can enhance the dissolution rate of poorly water-soluble drugs, therefore NCs serve as an effective and novel DDD system. In addition, NCs serve as efficient drug carriers for drugs with poor skin penetration capacity and poor water solubility; nonetheless, major drawbacks of NCs include regular dosing and size optimization [34]. As compared to conventional topical formulations, NC-based formulations provide rapid and enhanced skin penetration as well as boost skin deposition. In another study, it was observed that dexamethasone NCs greatly enhanced skin penetration as compared to dexamethasone-loaded ethyl cellulose nanocarriers and conventional dexamethasone creams [35]. NC-based formulations were found to provide rapid skin permeation and greater drug deposition in the dermis layer as compared to conventional nanocarriers in which most of the drugs remained in the epidermis layer [34,35].

Moreover, NCs provide numerous advantages, including greater therapeutic loading capacity, low excipient concentration, reproducibility, and facile method of preparation as compared to other nanocarriers, including ethosomes, niosomes, nanoemulsions, solid lipid nanoparticles, and polymeric nanoparticles. Limitations of lipid nanoparticles (such as solid lipid nanoparticles) include drug leakage and low entrapment efficiency because of their recrystallization tendency. Liposomes were found to be ineffective in penetrating deeper skin layers and might rupture the skin during permeation. On the other hand, niosomes are not suitable for transdermal purposes. In addition, niosomes are expensive, and their manufacturing process is complex. Therefore, the use of NC-based formulations is clearly beneficial for DDD because of their unique properties, including 100% drug loading, robust preparation method, pharmaceutical industrial scalability, and usage of small amounts of surfactant to ameliorate their stability [31].

## 4. Mechanism of Action of Dermal Nanocrystals

**Figure 2:**
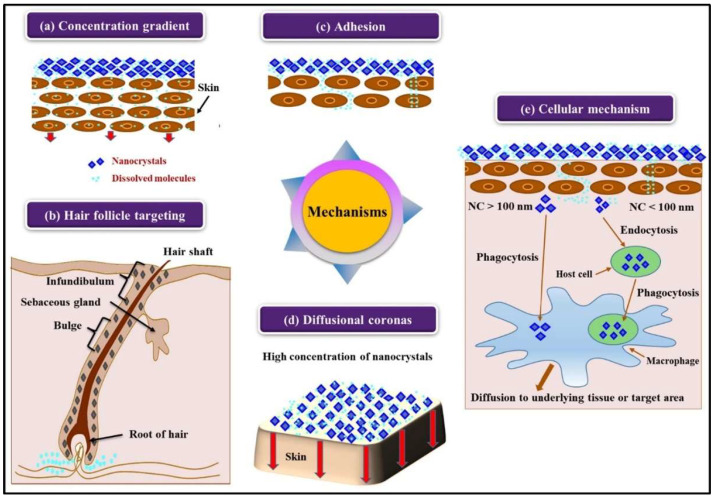
Mechanism of actions of dermal nanocrystals (NCs). (**a**) NCs exhibit enhanced passive diffusion through high-concentration gradients. (**b**) NCs can also penetrate through hair follicles. (**c**) NCs show an elevated adhesiveness to skin membranes. (**d**) NCs become encircled by diffusional coronas of the dissolved drug molecules. (**e**) Cellular mechanisms of NC delivery through phagocytosis or endocytosis. Reproduced with permission from Elsevier, Reference [25].

NCs have various unique features, including bioadhesion, improved permeation, and enhanced penetration through a membrane. These properties of NCs were utilized to develop oral and intravenous formulations. In addition, the use of NCs in DDD gained interest following the formulation of poorly soluble antioxidants, including hesperidin, apigenin, and rutin as nanosuspensions (NSs) for usage in anti-aging and skin protective products [14]. NCs are only admixed to the water phase of o/w lotions and dermal creams. In March 2007, the first products with rutin were launched in the market, for example, Juvedical products (age-decoder face fluid and face cream). Subsequently, Platinum Rare products containing hesperidin were made available in 2009 [14]. Formulations of rutin NCs were compared in vivo to a cream containing a water-soluble rutin derivative. The level of dissolved active ingredients in the water phase was 1/500 in the NC-based formulations. Still, NC-based formulations exhibited higher sun protection factors than water-soluble derivatives. NCs enhance the solubility of poorly soluble drugs in the water phase, which results in a higher concentration gradient between skin and formulation (Figure 2) that further leads to greater penetration than micron-sized powder. Rutin is typically more lipophilic. Thus, it shows better penetration as compared to hydrophilic derivatives. Furthermore, rutin may show more activities in the cells as compared to the derivatives. Active ingredients penetrated from the water phase into the skin are promptly replaced by rapidly dissolving active ingredients from the NCs; hence, they play a role as a depot in the water phase [14].

Indeed, this mechanism can be used to formulate topical products. When diclofenac sodium NS was delivered through the transdermal route in a Yucatan micropig skin model, it exhibited enhanced permeability flux of the drug across the skin by up to 3.8-fold than the control. Hesperetin NCs also showed significantly higher in vitro antioxidant properties in a free radical scavenging assay; thus, they can be utilized as an efficient adjuvant in topical or skin care preparations. In addition, the same NSs can also be used on mucosal surfaces as NS sprays or lotions. Positively charged polymers can be used as stabilizing agents for the drug NCs to enhance their adhesion properties. It has been observed that the opposite charge results in an enhanced affinity of the drug NCs to the negatively charged cells. Previously, this theory was demonstrated by generating antiseptic lipid nanoparticle sprays by utilizing cetylpyridinium chloride as an antiseptic and cationic surfactant. By using the combination technology, ultrafine lidocaine base NCs were produced as extended-release formulations for topical use. Administration through rectal and vaginal routes can be used to treat local sexually transmitted diseases by evenly spreading the drug in the local area [14].

## 5. Advantages of Using Nanocrystal-Based Formulations in Enhancing Dermal Drug Delivery

### 5.1. Increased Drug Loading Capacity

Drug loading might significantly affect both the processability and qualities of end products; thus, it is important to develop dosage forms. NCs show high loading capacity as the drug itself is utilized as the delivery system, along with a small portion of amphiphilic block copolymers or surfactants. Studies confirmed that trans-resveratrol nano-suspension had a drug loading capacity of 28.1%, while for mixed micelles, it was only 8.4% [36,37]. Celastrol is a powerful antioxidant that might trigger apoptosis of cancer cells by suppressing the functions of the proteasome. Nonetheless, this drug shows poor water solubility and poor oral bioavailability. Interestingly, NS of celestrol increased the drug loading capacity to 86.83%; nanoscale exosomal formulation exhibited only 20% drug loading capacity [38].

### 5.2. Easier and Deeper Penetration into the Skin Tissue

An elevated level of the total surface area of NCs also increases their compatibility and easier penetration through the skin tissues either directly or through the hair follicles, which eventually improves drug utilization [39]. Therefore, it is important to determine the optimal size of different NCs for effective skin penetration. It was observed that quercetin NCs and baicalin NCs showed greater solubility and stability as compared to microcrystals, which indicates the importance of smaller particle sizes on the release pattern of NCs. In a study, different sizes of curcumin NCs, including 60  nm, 120  nm, and 480  nm, were analyzed, where smaller particle sizes containing curcumin NCs showed higher drug accumulation in the skin layer, which further demonstrates that smaller particle sizes show greater physicochemical properties [40]. Nonetheless, not all smaller-size drug NCs exert superior effects in DDD. For example, serum level kinetics of caffeine NCs showed that 694  nm NCs exhibited higher concentration than 204  nm NCs after 20  min; however, 204  nm NCs enhanced the bioavailability of caffeine at early time points [21]. In general, particle size distributions for NCs are directly linked with the drug administration effect; therefore, proper mixing or selection of different particle sizes for NCs might mediate greater pharmacokinetic properties. In addition, NCs have the capacity for deeper penetration into the skin via passively diffusing through the keratinocytes or natural channels of skin appendages. The high drug-loading capacity of NCs also mediates enhanced cosmetic effects at the target site. Thus, NCs have great potential in cosmeceuticals and cosmetics [41].

### 5.3. Increased Passive Diffusion

**Figure 3:**
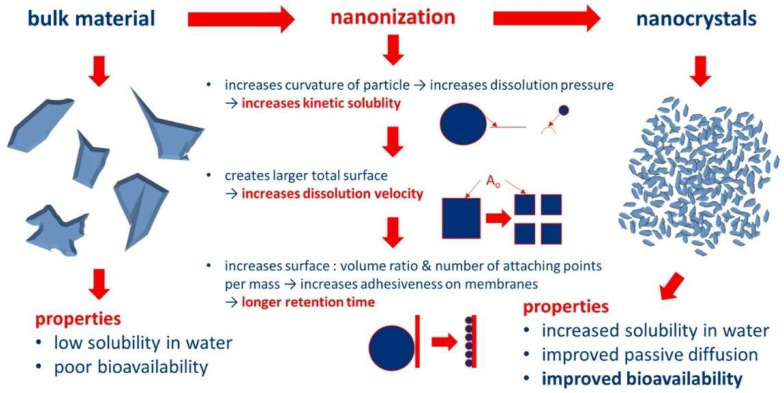
Schematic diagram showing enhanced properties of NCs after modification. Reproduced with permission from Elsevier, Reference [21].

NCs exhibit higher dissolution velocity and saturation solubility because of their smaller particle size and larger surface area (Figure 3). Enhanced passive diffusion takes place because of this concentration gradient between the skin membrane and topically applied formulation [41]. Furthermore, it was observed that the liquid in the meniscus played a role as a reservoir for dissolved drug molecules from which a constant curcumin penetration might occur into the skin while studying the increase in transdermal and dermal transmission of curcumin by NCs and smart films. On the other hand, drugs that have already crossed the meniscus and left its liquid reservoir might be substituted by molecules that were secreted from the curcumin particles. Hence, a gradient of highly concentrated dissolved curcumin was established and maintained between the SC and aqueous meniscus. The concentration gradient could also be affected by the amount of dissolved activity in the carrier, which may change because of the dispersion media and/or the drug particle sizes [41].

## 6. Nanocrystal Preparation Techniques

**Figure 4:**
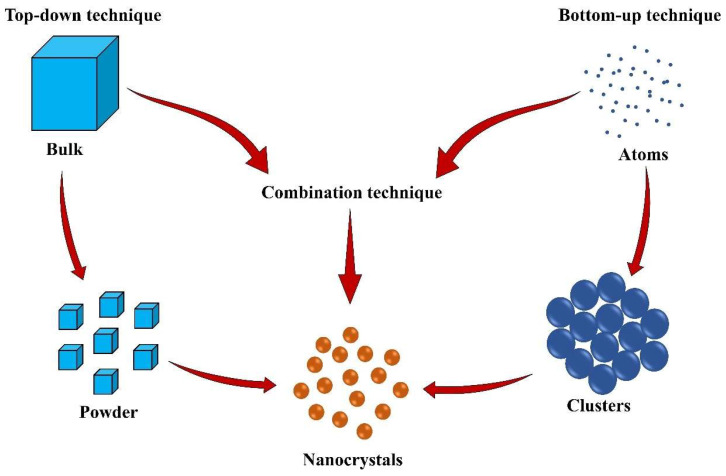
Preparation methods for nanocrystals.

NCs are typically prepared either through bottom-up techniques (by altered crystallization/antisolvent precipitation) or top-down techniques (by mechanical breakage/attrition of a crystalline powder) (Figure 4). Although top-down techniques are widely used in commercial applications, these methods have some inherent disadvantages (Table 1) that require the development of alternative methods. On the other hand, bottom-up techniques are not fully established for commercial applications; nonetheless, it has greater potential to generate small-size drug NCs while requiring less energy.

### 6.1. Top-Down Techniques

#### 6.1.1. Milling

Most of the NC products that are available in the market are prepared by using wet ball milling (WBM) [42]. WBM method uses mechanical attrition to wet particles by using an aqueous solution of surfactants and shearing as well as ground via using milling balls in a milling container. WBM results in reduced particle sizes, which might reach a few hundred micrometers; however, the traditional milling process can be modified to generate NCs [42]. Furthermore, the manufacturing process can be reproducible. Major drawbacks of this manufacturing process include decreased crystallinity, long operation time, high energy input, and contamination from erosion of metal milling pearls or balls [42]. However, polymeric beads can be used to minimize the contamination and erosion. On the other hand, jet milling is used to manufacture microparticles without using organic solvents. A major advantage of using solvent-free jet milling is the prevention of toxicity because of the very short production time and the absence of organic solvents [43].

#### 6.1.2. High-Pressure Homogenization (HPH)

In a chamber, two fluid streams of particle suspensions collide under high pressure in the case of HPH, which results in the collision of particles following particle rupture. In piston-gap homogenizers, nanosized solid particles are manufactured by forcing a suspension of drug particles with a piston by a thin gap under high pressure. The turbulent flow and high shear forces break the particles, and the particle outcome is determined by the number of piston-moving cycles, particle hardness, and the power of homogenization. Nevertheless, HPH needs complex equipment, high-energy input, and high process temperature, which may experience a possible breakdown of the components and might have less yield as compared to wet milling [43].

#### 6.1.3. Laser Ablation (LA)

LA is comparatively a newer process for NC preparation. In the case of LA, a laser beam is focused on a solid target to eject materials. Subsequently, the ejected materials form nanoparticles in the surrounding liquid. The mixed suspensions of microparticles are then fragmented into nano-sized particles through laser-induced fragmentation [44]. Depending on the duration of the laser pulse, LA can be grouped into femtosecond, picosecond, and nanosecond laser irradiation. Several factors affect the particle sizes, including properties of the suspension, scanning speed, and laser intensity. No organic solvent is involved in LA; however, a small fraction of the drug might go through oxidative degradation and alterations in crystal states because of extreme power. LA has already been utilized in the preparation of curcumin, megestrol acetate, and paclitaxel NSs [45,46].

#### 6.1.4. Ultrasound

In the case of ultrasound, drug particles are fragmented into smaller particles by the vibration of acoustic waves. Nucleation can be enhanced through ultrasound via the generation of acoustic cavitation in solution and rapid dispersal of the drug solution. This method is highly reproducible and can easily be operated in laboratory settings. Ultrasound-guided precipitation of nanoparticles changes agglomeration, growth, nucleation, and mixing processes [47]. Various factors affect the size of NCs, including the cavitation depth, horn immersion depth, horn length, and ultrasound treatment [45,48].

### 6.2. Bottom-Up Techniques

#### 6.2.1. Control Flow Cavitation (CFC)

CFC makes the best process condition for NC preparation after regulating the density, location, size, and implosion intensity of bubbles in the cavitation zone. The controlled energy released by the microbubble implosions and the capacity to regulate the energy of cavitation are the desired distributions of particle sizes to be achieved [49]. In addition, to generate micro- and nanostructured materials, CFC turns destructive force into constructive with high-intensity energy force [49]. CFC is a highly effective and scalable technique with outstanding reproducibility and excellent process control [43,50].

#### 6.2.2. High Gravity-Controlled Precipitation (HGCP)

HGCP is the improved version of the precipitation method that uses gravity regulation to prepare smaller and more uniform drug NCs. There are several factors that influence particle size, including volumetric flow rate, rotational speed, and reactant concentration. The drug suspension in the device can be circulated for long-term reaction and mixing. Nevertheless, continuous nucleation is achieved because of the local oversaturation of the feed stream at the turbulent edge during mixing, which limits the use of the HGCP method in industrial applications [51]. So far, HGCP has been effectively utilized in laboratory-scale preparation of sorafenib and salbutamol sulfate [45,52].

#### 6.2.3. Spray Drying

Spray drying is a single-step method for the conversion of pastes, slurries, suspensions, emulsions, and solutions into powders in a continuous manner. This method also permits the generation of particles with controlled morphological aspects and sizes. However, the conventional spray drying method is limited to the generation of particles with sizes between 2 μm and 5 μm [43].

#### 6.2.4. Microfluidization

The microfluidization method has been developed on the basis of the dynamics of carefully designed microchannels through which liquids go through high-pressure collision up to 1700 bar (25,000 psi) [53]. The active chemicals and lipids are mixed by the compressed air-driven pump at very high velocities in the selected microchannels, which further generates stable nano-sized delivery systems ranging from 50 to 100 nm [54]. At present, single-step dual-channel and two-step single-channel microfluidizers are commonly used in industries. The use of a single-step dual-channel microfluidizer is more advantageous than the two-step single-channel microfluidizer since it consumes less energy and less expensive oil and lipid materials utilized in the preparation of NCs [55,56,57,58,59].

#### 6.2.5. Liquid Antisolvent Precipitation (LAP)

In order to generate NCs, the LAP method mixes a solution stream (organic phase) dissolved with an insoluble drug along with an aqueous antisolvent. Most commonly, the solution–antisolvent technique is used for nanoprecipitation. This method is cost-effective and simple as it contains only nucleation and growth steps [48]. There are two steps for preparing optimized NCs. Nevertheless, LAP involves the recrystallization of unstable crystal particles, which results in precipitation and aggregation of NCs [60]. Moreover, the problem of solvent residues arises due to the usage of organic solvents in the manufacturing process; therefore, LAP is not suitable for drugs that are neither insoluble in non-aqueous nor soluble in aqueous solvents. The LAP method has previously been used to prepare suspensions of budesonide and hydrochlorothiazide [45].

### 6.3. Combination Technique

The combination technique involves the use of any of the two above-mentioned techniques. Various combinations, including HPH followed by milling, spray-drying, and further processed by homogenization or milling, freeze-drying followed by milling, and precipitation followed by HPH are some combination techniques that are commonly utilized to obtain better production and product benefits [61]. Table 2 shows a number of patented topical NCs and production techniques.

**Table 1:** Comparative advantages and disadvantages of top-down and bottom-up approaches for nanocrystal production.

Preparation Method	Advantages	Disadvantages	References
Top-down techniques			
Milling	Reduces particle size in a reproducible manner	Decreased crystallinity, long operation time, high energy input, and contamination from erosion of metal milling pearls or balls	[42]
High-pressure homogenization	Shorter production times; absence of organic solvents; ease of scale-up	Needs complex equipment, high-energy input, and high process temperature, which may experience possible breakdown of the components and might have less yield	[43]
Laser ablation	No organic solvent is required	A small fraction of the drug might go through oxidative degradation and alterations in crystal states because of extreme power	[45,46]
Ultrasound	Highly reproducible; easy operation in laboratory settings; alters agglomeration, nucleation, and mixing process	Low scalability; very high ultrasound energy can increase collisions, agglomerations, and sizes of particles	[47]
Bottom-up techniques			
Controlled flow cavitation (CFC)	Highly effective and scalable technique with outstanding reproducibility and excellent process control	-	[43,50]
High gravity-controlled precipitation	Generates smaller and more uniform drug NCs; drug suspension in the device can be circulated for long-term reaction and mixing	Continuous nucleation is achieved because of the local oversaturation of the feed stream at the turbulent edge during mixing, which limits the use of this method in industrial applications	[51]
Spray drying	Single-step method for the conversion of pastes, slurries, suspensions, emulsions, and solutions into powders in a continuous manner; permits the generation of particles with controlled morphological aspects and sizes	Limited to the generation of particles with sizes between 2 μm and 5 μm	[43]
Microfluidization	Generates stable nano-sized delivery systems ranging between 50 and 100 nm	A two-step single-channel microfluidizer requires more energy and more expensive oil and lipid materials	[54,55,56,57,58,59]
Liquid antisolvent precipitation	Cost-effective and simple manufacturing process	Recrystallization of unstable crystal particles, which results in precipitation and aggregation of NCs	[48,60]

**Table 2:** A summary of patented topical nanocrystals and production techniques.

Topical Nanocrystals	Topical Uses	Production Techniques	Particle Size	Features	Patent Number	Patent Year	References
Rutin	Anti-aging effects on the skin	Combination technique: low-energy pearl milling followed by a high energy high-pressure homogenization (HPH)	604–820 nm	Enhanced bioactivity of rutin in the skin	US9114077B2	2015	[31,62]
Silver sulfadiazine	Prevents wound infections in individuals with burns	Milling	150–500 nm	Silver sulfadiazine nanocrystals were superior to conventional cream preparations	US9572777B2	2017	[31,63]
Zinc oxide	Prevents or treats skin irritation including diaper rash, burns, or cuts	Milling	5–30 nm	Enhanced antimicrobial activity exhibited by finely divided transparent zinc oxide with no unwanted white coating on the skin at lower concentration as compared to ordinary commercially available zinc oxide	WO1994007509A1	1992	[31,64]
Spironolactone	Exerts anti-androgenic properties to treat hirsutism, acne, rosacea or androgenic alopecia	HPH	400–600 nm	Spironolactone release rate from the nanosuspension (NS) was markedly faster as compared to the aqueous cream	US8003690B2	2011	[31,65]
Calcipotriol monohydrate	Treats psoriasis	HPH	200–600 nm	Release rate of calcipotriol NSs was significantly higher than the calcipotriol ointment	EP2515874B1	2014	[31,66]
Zinc oxide and titanium dioxide	Sunscreen	Milling	5–150 nm	Exhibited improved and balanced absorption of ultraviolet A and B radiations	EP0535972B1	1995	[31,67]
Paclitaxel	Treats psoriasis	Milling	100 nm to 1500 nm	Showed enhanced skin penetration at low concentration and caused less skin irritation	WO/2017/049083	2017	[31,68]

## 7. Applications of Nanocrystal-Based Formulations with Enhanced Dermal Drug Delivery in the Treatment of Skin Disorders

### 7.1. Melanoma

Melanoma is a type of skin cancer that involves malignant neoplasm of the melanocytes [69]. Melanocyte produces melanin (the natural skin pigment), and they are located in the basal layer of the epidermis [70,71]. Noscapine is a phthalide isoquinoline alkaloid derived from the opium poppy. This alkaloid was found to prevent the proliferation of cells and mediate apoptosis in B16LS9 melanoma cells. However, noscapine suffers from various problems, including poor water solubility as well as poor oral bioavailability. Noscapinoids (synthetic derivatives of noscapine) were incorporated in silver NCs to overcome these drawbacks because silver decreases the extent of UV-induced DNA damage and apoptosis. The combined effects of noscapinoids and silver were assessed in B16-F1 cells through cellular uptake assay, apoptosis assay, and cell proliferation assay (Table 3). The study findings suggested that noscapinoids bearing silver NCs exhibited better apoptosis, cellular uptake, and cytotoxicity in the cell line [31,72]. In a study, a combination of UV-B absorbing quercetin (a natural antioxidant flavonoid) and titanium dioxide was incorporated into a nanogel to prevent skin cancer [73]. It was observed that 70% of drug release in 24 h was observed with the quercetin and titanium dioxide nanogel. In addition, this nanogel downregulated cyclin D1, proliferating cell nuclear antigen, EP3, and cyclooxygenase-2, as well as limited the cell cycle and inflammatory pathways than quercetin alone [73]. Collectively, these findings indicate the potential of this nanogel as an effective DDD system to prevent UV-B radiation-induced skin cancer [31,74].

### 7.2. Pain and Inflammation

Meloxicam is a nonsteroidal anti-inflammatory drug (NSAID) used in the treatment of acute pain and inflammation; nonetheless, this drug suffers from poor solubility, which leads to the prolonged onset of action that hinders its development [75,76]. Meloxicam NCs were effectively manufactured by nanoprecipitation, which resulted in greater transdermal permeability and over double the elevation in plasma level area under the curve over 24  h as compared to normal formulation [77]. Collectively, the DDD of meloxicam NCs was found to be beneficial [41]. Curcumin is an effective and natural anti-inflammatory agent [78], and its NCs were developed owing to its poor solubility in water and oil. Skin-friendly curcumin NCs with an average particle size of around 200  nm were developed by using Plantacare stabilizers and the smartcrystal technique [40]. In a study, analgesic and anti-inflammatory activities of a nanogel of flurbiprofen (an NSAID) NCs were evaluated in rat models. Stabilizer, Plantacare 2000 (BASF, Ludwigshafen, Germany) and the wet milling method were used to generate flurbiprofen NS. The developed flurbiprofen NS was further mixed with various carrier gels, such as hydroxypropyl methylcellulose (HPMC), polycarbophil, oleogel, and chitosan. Flurbiprofen NS HPMC gel was found to exert greater anti-inflammatory effects as compared to physical mixture gel and coarse suspension gel. In terms of analgesic properties, flurbiprofen NCs exhibited a higher latency period (180  min) than the control group. Therefore, the HPMC gel elevated the NS contact duration with skin and mediated DDD, while nanosized particle-based NSs and a wider surface area enhanced the pharmacodynamics of flurbiprofen in vivo [79].

Researchers also developed two NC preparations of diclofenac (an NSAID) crystal forms to increase the topical bioavailability. Diclofenac NCs with particle sizes varying from 279 to 315  nm showed better DDD as well as enhanced skin penetration and deposition compared to commercial topical formulations and coarse suspensions of diclofenac sodium. Collectively, these findings indicate the importance of the sizes of NCs in drug solubility, henceforth, the capacity of poorly soluble drugs to penetrate the epidermis and deposit in the deeper skin layers [80]. In a different study, the effectiveness of curcumin NC particle size of 300  nm in terms of skin and hair follicle penetration was assessed via the ex vivo pig ear method [81]. These NCs were found to be effective in hair follicle targeting and skin penetration [81]. In another study, NCs of 18β-Glycyrrhetinic acid, a pentacyclic triterpene that occurs in plants, were prepared by HPH to treat inflammatory skin disorders [82]. These NCs were characterized by photon correlation spectroscopy (PCS), scanning electron microscopy, thermogravimetric analysis, and X-ray diffractometry. The average particle size of 18β-Glycyrrhetinic acid NCs was 552.0  ±  9.8  nm, and the polydispersity index (PI) was 0.13–0.10. The solubility and skin penetration capacity of these NCs were greatly increased after nano crystallization. However, thermal stability and crystallinity were decreased [82,83].

### 7.3. Psoriasis

Psoriasis, an autoimmune condition, is a chronic and inflammatory skin condition. As DDD involves lower chances of systemic adverse reactions caused by the drug, it is the preferred route for mild-to-moderate psoriasis management [84]. Researchers prepared NCs of apremilast (an immunosuppressive drug) by using the wet milling method to enhance its DDD. Apremilast NCs exhibited two times greater saturation solubility than the micronized apremilast. As compared to oral apremilast preparations, apremilast NCs were found to increase the skin distribution of apremilast to treat psoriasis without adverse effects [85]. In a study, methotrexate (an anti-metabolite) NCs were prepared by using the bottom-up technique to target and maintain its release at the skin application site [86]. The average particle size of methotrexate NCs was 678  ±  15 nm, which exhibited prolonged in vitro drug release over a 72 h period. In addition, methotrexate NCs were also incorporated into the shafts of disintegrating microneedles arrays with a drug loading of 2.48 mg per array, which resulted in the deposition of 25.1% of the packed methotrexate NCs in the epidermis. Collectively, these findings indicate the potential of methotrexate in effective targeting and providing prolonged intradermal delivery to treat psoriasis [83,86].

### 7.4. Acne Vulgaris

Acne vulgaris is an inflammatory skin condition of the pilosebaceous follicle. In a study, azelaic acid (a saturated dicarboxylic acid) NC hydrogel composed of Pluronic F-127 and hyaluronic acid delivered azelaic acid into the SC and inner skin layer to treat acne and rosacea [87]. It was observed that the hydrogel prepared with 15% Pluronic F-127, 1% hyaluronic acid, and 10% lyophilized NC azelaic acid solution showed effective rheological properties and drug delivery required for an in situ gelling framework for skin exposure [87]. Tretinoin (a vitamin A derivative) NCs were developed by precipitation method in a different study to increase the cutaneous targeting of tretinoin and to enhance photostability. These NCs were characterized by using transmission electron microscopy (TEM) and photon correlation spectroscopy. On the other hand, the photodegradation of tretinoin NCs was analyzed by the UV irradiation method, which revealed that tretinoin NCs showed greater photostability. Furthermore, these NCs also showed increased cutaneous distribution [83,88,89].

### 7.5. Bacterial Infections

Topical antibiotics are used to treat various bacteria-caused infections [90]. However, there is a need for the development of novel therapeutic approaches to cure skin infections because of the emergence of bacterial antibiotic resistance. In a study, researchers developed fusidic acid (antistaphylococcal antibiotic) NCs by using the nanoprecipitation method to treat skin infections [91]. Since fusidic acid shows low water solubility, researchers converted them into NCs to improve its saturation solubility, local bioavailability, and effectiveness for DDD. In addition, lyophilized fusidic acid NC was also incorporated into a cream preparation, which was found to result in a rapid drug diffusion from the cream formulation. As compared to commercial cream, fusidic acid NC cream exhibited better in vivo antibacterial properties and wound healing [91]. In another study, NCs of nitrofurazone (a topical antibiotic) were prepared by using the wet milling method, which was then converted into gels for extensive assessment. After the gel formation by using carbopol formulation, a scanning electron microscope (SEM) showed that nitrofurazone formed rectangular-shaped particles. It was revealed by in vitro studies that nitrofurazone dissolution from nanogel was significantly greater (85.73%) after 24  h than the nitrofurazone-marketed gel (70.55%). Furthermore, as compared to the nitrofurazone-marketed gel (61.30  g/cm^2^), the quantity of nitrofurazone that penetrated skin was significantly higher with the nitrofurazone nanogel (220.89  g/cm^2^) [83,92]. Silver sulfadiazine (a topical antibiotic) NCs were prepared by using the wet milling method. These NCs showed enhanced dissolution rate and antibacterial effect in vitro; however, they suffered from major problems, including poor physical stability and high cytotoxicity. In order to overcome these problems, silver sulfadiazine NCs were gradually encapsulated within chitosan hydrogels. Silver sulfadiazine NCs loaded hydrogels showed enhanced antibacterial properties in vitro owing to the larger contact area along with low cytotoxicity. On the other hand, it was revealed by in vivo studies that the quickest healing time, optimum collagen deposition, and nearly full re-epithelialization were observed in wounds cured by silver sulfadiazine NCs loaded hydrogels [83,93].

### 7.6. Fungal Infections

Fungal infections are responsible for causing over 1.5 million deaths every year; therefore, strong research is required in this field to develop novel therapeutics [94]. In a study, luliconazole (a topical antifungal agent) NCs were developed by using the nanoprecipitation method and then incorporated into a hydrogel to increase the dissolution and antifungal properties. Luliconazole NCs showed increased skin absorption and drug retention. In addition, luliconazole NCs showed greater antifungal action than conventional luliconazole, along with 5-fold greater solubility and 4-fold increased dissolution. Luliconazole hydrogel also caused negligible irritation in Wister rats [95]. In another study, nanogel of the clotrimazole (a topical broad-spectrum antifungal agent) NCs were prepared by using media milling to treat fungal infections [96]. These researchers used polysorbate 80 (a non-ionic stabilizer) at a 1.5% *w*/*v* concentration to reduce the particle sizes from micrometer to nanometer range, and the PI was 0.211. Clotrimazole NCs were then combined with carbopol 934 polymer to generate a nanogel with a viscosity of 85.43  ±  3.06 Pascal-second at 25  °C, which made clotrimazole NCs easier to apply on the skin. Moreover, clotrimazole NCs effectively treated cutaneous Candida albicans in Wister rats [83,96].

### 7.7. Eczema

Eczema or atopic dermatitis is a skin condition that causes inflamed, dry, and itchy skin [97]. Beclomethasone (a corticosteroid) NCs were prepared in a study by using the antisolvent nanoprecipitation method to treat eczema. These NCs showed 745.5 times greater saturation solubility than even pure beclomethasone dipropionate. The local skin accumulation efficiency of beclomethasone NCs was 6.20, which was significantly higher than the local skin accumulation efficiency of 0.25 of the brand formula [83,98].

### 7.8. Skin Aging

Skin aging is a complicated process where skin deteriorates with age because of the synergistic effects of environmental factors, hormonal deficiency, photo-aging, and chronological aging [83,99,100]. In a study, caffeine NCs were prepared by HPH or the pearl milling method to prevent fiber formation and crystal development, as supersaturation tends to generate medium solubility drugs. These NCs were developed based on the low-energy milling method along with ideal stabilizers and reduced dielectric constant dispersion media. In addition, polyvinylpyrrolidone (PVP-40), carbopol 981, and tween 80 were used to generate caffeine NS. Collectively, caffeine NCs showed enhanced skin permeation, skin protection, and also anti-ageing [101].

### 7.9. Herpes Simplex Virus (HSV) Infections

HSV can cause infections in different parts of the human body, most commonly the mouth and genital areas [102]. Researchers developed acyclovir (an antiviral drug) NCs via wet media milling in a high-pressure homogenizer to enhance DDD. These NCs were found to be crystalline naturally and had particle sizes ranging from 450 to 550  nm. Saturation solubility of acyclovir NCs was 1.6-fold higher than acyclovir in micronized form. Both NS and nano cream of acyclovir showed 6.3-fold and 2.4-fold higher skin permeation than the conventional preparations, respectively [83,103].

### 7.10. Skin Manifestations of Tick Bites

Ticks can play a role as a vector of disease-causing agents in humans. Azithromycin (a macrolide antibiotic) NCs were prepared to control skin infection after tick bites [104]. Azithromycin NCs were found to be stable at 4  °C for a year. PCS, laser diffraction, and light microscope were used to characterize the azithromycin NCs, which revealed that these NCs were in the nanometer range. Saturation solubility of azithromycin NCs in water was up to 3-fold higher than the raw drug powder; henceforth, these NCs showed enhanced skin bioavailability [83,104].

### 7.11. Frostbite-Related Infections

Individuals with frostbite are at risk of wound infections caused by bacteria. A nanogel loaded with Ganoderma lucidum (a medicinal mushroom) NCs was manufactured by researchers through the HPH method for DDD to treat frostbite [105]. Particle size analysis and SEM demonstrated that Ganoderma lucidum NS retained their particle sizes after formulating gel with carbopol. Ganoderma lucidum nanogel was found to increase the cumulative amount of Ganoderma lucidum in the upper skin layer 6-fold greater than Ganoderma lucidum carbopol gel, along with no edema or erythema. Moreover, Ganoderma lucidum nanogel showed better therapeutic activity than the Ganoderma lucidum carbopol gel [83,105].

**Table 3:** A summary of nanocrystal-based formulations with enhanced dermal drug delivery in the treatment of skin disorders.

Drug Nanocrystals	Skin Disorder	Preparation Method	Study Outcome	References
Noscapinoids bearing silver nanocrystals (NCs)	Melanoma	Precipitation	Noscapinoids bearing silver NCs exhibited better apoptosis, cellular uptake, and cytotoxicity in B16-F1 cells	[72]
Meloxicam NCs	Pain and inflammation	Nanoprecipitation	Meloxicam NCs showed greater transdermal permeability and over double the elevation in plasma level area under the curve over 24 h as compared to normal formulation	[77]
Flurbiprofen NCs	Pain and inflammation	Wet milling	Flurbiprofen nanosuspension (NS) gel showed greater anti-inflammatory effects as compared to physical mixture gel and coarse suspension gel	[79]
Diclofenac NCs	Pain and inflammation	Wet milling	Diclofenac NCs with particle sizes varying from 279 to 315 nm showed better DDD as well as enhanced skin penetration and deposition compared to commercial topical formulations and coarse suspensions of diclofenac sodium	[80]
18β-Glycyrrhetinic acid NCs	Pain and inflammation	High-pressure homogenization (HPH)	Solubility and skin penetration capacity of these NCs were greatly increased after nano crystallization	[82]
Apremilast NCs	Psoriasis	Wet milling	Apremilast NCs exhibited two times greater saturation solubility than the micronized apremilast	[85]
Methotrexate NCs	Psoriasis	Acid–base neutralization precipitation	Methotrexate NCs were effective in effective targeting and providing prolonged intradermal delivery to treat psoriasis	[86]
Azelaic acid NC hydrogel	Acne vulgaris	Milling	Showed effective rheological properties and drug delivery required for an in situ gelling framework for skin exposure	[87]
Tretinoin NCs	Acne vulgaris	Precipitation	Tretinoin NCs showed greater photostability and increased cutaneous distribution	[88]
Fusidic acid NCs	Bacterial infections	Precipitation	Fusidic acid NC cream exhibited better in vivo antibacterial properties and wound healing	[91]
Nitrofurazone NCs	Bacterial infections	Wet milling	Nitrofurazone dissolution from nanogel was significantly greater (85.73%) after 24 h than the nitrofurazone-marketed gel (70.55%)	[92]
Silver sulfadiazine NCs loaded hydrogels	Bacterial infections	Wet milling	Silver sulfadiazine NCs loaded hydrogels showed enhanced antibacterial properties in vitro owing to the larger contact area along with low cytotoxicity, while in vivo studies revealed that the quickest healing time, optimum collagen deposition, and nearly full re-epithelialization were observed in wounds cured by silver sulfadiazine NCs loaded hydrogels	[93]
Luliconazole NCs	Fungal infections	Precipitation	Luliconazole NCs showed greater antifungal action than conventional luliconazole along with 5-fold greater solubility and 4-fold increased dissolution	[95]
Clotrimazole NC nanogel	Fungal infections	Media milling	Clotrimazole NCs effectively treated cutaneous *Candida albicans* in Wister rats	[96]
Beclomethasone NCs	Eczema	Precipitation	These NCs showed 745.5 times greater saturation solubility than even pure beclomethasone dipropionate. Local skin accumulation efficiency of beclomethasone NCs was 6.20, which was significantly higher than the local skin accumulation efficiency of 0.25 of the brand formula	[98]
Caffeine NCs	Skin aging	High-pressure homogenization	Caffeine NCs showed enhanced skin permeation, skin protection, and anti-ageing	[101]
Acyclovir NCs	Herpes simplex virus infections	Media milling	Saturation solubility of acyclovir NCs was 1.6-fold higher than acyclovir in micronized form	[103]
Azithromycin NCs	Skin manifestations of tick bites	Bead milling	Saturation solubility of azithromycin NCs in water was up to 3-fold higher than the raw drug powder, henceforth these NCs showed enhanced skin bioavailability	[104]
Nanogel loaded with *Ganoderma lucidum* NCs	Frostbite-related infections	High-pressure homogenization	*Ganoderma lucidum* nanogel was found to increase the cumulative amount of *Ganoderma lucidum* in the upper skin layer 6-fold greater than *Ganoderma lucidum*-carbopol gel, along with no oedema or erythema	[105]
Glabridin NCs	Hyperpigmentation	Anti-solvent precipitation-homogenization	The developed nano formulation significantly ameliorated the penetration of glabridin through rat skin without lag phase in both in vitro and in vivo	[83]
Diosmin NCs loaded sodium alginate wafers	Diabetic foot ulcer	Antisolvent precipitation	These NC-loaded wafers exhibited high porosity, mucoadhesion properties, and prolonged degradation time	[31]

### 7.12. Hyperpigmentation

Hyperpigmentation is a common skin condition that involves the appearance of darker patches on the skin [106]. In a study, glabridin (an important flavonoid extracted from licorice root) NCs were developed to enhance drug penetration through the skin. These researchers prepared glabridin NSs by using an anti-solvent precipitation–homogenization process. The average particle size of the NSs was 149.2 nm, and the PI was 0.254. The developed nano formulation significantly ameliorated the penetration of glabridin through rat skin without lag phase in both in vitro and in vivo. In a different study, cellulose NCs with a particle size of 310  nm were measured by dynamic light scattering. The researchers incubated hydroquinone (a skin-lightening agent) and cellulose NCs mixture to generate a combination of cellulose hydroquinone NCs. This combination resulted in a sustained release of hydroquinone [83,107].

### 7.13. Diabetic Foot Ulcer

Diabetic foot ulcer is a major complication of diabetes mellitus, which can further lead to hospitalization and even amputation of the lower limb if not treated in a timely manner. Diosmin is a natural compound found in some plants that shows strong antiulcer, anti-inflammatory, anticancer, and antioxidant properties. Diosmin NC-loaded sodium alginate wafers were prepared to treat diabetic ulcers in rats. These NC-loaded wafers exhibited high porosity, mucoadhesion properties, and prolonged degradation time. Curcumin is a good antimicrobial agent that shows no toxicity, and nanocellulose is widely used in wound dressing since it shows good compaction properties as well as excellent absorbing ability. Therefore, curcumin-loaded nanocellulose NCs were developed to increase the regeneration of hair follicles and sebaceous glands via confining bacterial growth [31,108].

## 8. Future Perspectives and Challenges

Skin barriers appear as a major barrier to the formulation of topical drugs. Treatment of skin disorders requires suitable and effective drugs delivered through the skin. Surprisingly, despite years of efforts in the improvement of DDD, drugs delivered through the skin experience very low bioavailability. In addition, a major problem with most of the marketed dermal therapy products is systemic drug absorption, which further results in systemic toxicity. Therefore, there is a growing interest in the development of nanocrystal-based formulations for DDD owing to their increased surface area, enhanced adhesion property, maximum drug loading capacity, and increased solubility. Furthermore, this growing technology offers some added advantages, including the combination with conventional dosage forms and the transformation of NC suspension into a gel for easier application. There are a range of top-down and bottom-up techniques to prepare NCs that mainly aim at reducing the sizes of drug particles. The skin permeation process is further enhanced by the localized drug reservoir, which further leads to the creation of a higher level of drug load in the hair follicles and skin furrows. Thus, an extended drug release is obtained through the diffusion of drug NCs from the hair follicles in a concentration-dependent manner [109]. Indeed, the DDD of NCs provides exciting solutions to numerous problems of conventional topical preparations. Nonetheless, although NCs offer numerous therapeutic benefits, there are some drawbacks of NC-based formulations in the case of DDD. These drawbacks include the requirement for more frequent administration of NCs [33], the possibility of the development of flocculation and agglomeration of NCs [110], and greater risks of systemic absorption as well as systemic adverse reactions [33]. Therefore, more studies are required to explore ways of overcoming these challenges and for the commercialization of nanocrystal-based formulations for DDD.

## 9. Conclusions

In terms of DDD, the easy preparation methods of NCs make them a preferred choice for screening during the initial and discovery phases to assess the presence of any correlation between the bioavailability and particle size of a drug. So far, a range of dermal NCs have been developed in various studies that have been evaluated in terms of their skin penetration capacity. Findings from these studies demonstrate that NCs can increase the skin penetration capacity of poorly soluble drugs in a size-dependent manner, where small-sized NCs showed enhanced and deeper skin penetration. Moreover, enhanced skin penetration by the drug is more likely to be achievable when a higher number of NCs come into direct skin contact.

## Data Availability

The data presented in this study are contained within this article.
